# Evaluation of the phenotypic and genomic background of variability based on litter size of Large White pigs

**DOI:** 10.1186/s12711-021-00692-5

**Published:** 2022-01-03

**Authors:** Ewa Sell-Kubiak, Egbert F. Knol, Marcos Lopes

**Affiliations:** 1 Department of Genetics and Animal Breeding, Poznan University of Life Sciences, Poznań, Poland; 2 Topigs Norsvin Research Centre, Beuningen, The Netherlands; 3 Topigs Norsvin, Curitiba, Brazil

## Abstract

**Background:**

The genetic background of trait variability has captured the interest of ecologists and animal breeders because the genes that control it could be involved in buffering various environmental effects. Phenotypic variability of a given trait can be assessed by studying the heterogeneity of the residual variance, and the quantitative trait loci (QTL) that are involved in the control of this variability are described as variance QTL (vQTL). This study focuses on litter size (total number born, TNB) and its variability in a Large White pig population. The variability of TNB was evaluated either using a simple method, i.e. analysis of the log-transformed variance of residuals (LnVar), or the more complex double hierarchical generalized linear model (DHGLM). We also performed a single-SNP (single nucleotide polymorphism) genome-wide association study (GWAS). To our knowledge, this is only the second study that reports vQTL for litter size in pigs and the first one that shows GWAS results when using two methods to evaluate variability of TNB: LnVar and DHGLM.

**Results:**

Based on LnVar, three candidate vQTL regions were detected, on *Sus scrofa* chromosomes (SSC) 1, 7, and 18, which comprised 18 SNPs. Based on the DHGLM, three candidate vQTL regions were detected, i.e. two on SSC7 and one on SSC11, which comprised 32 SNPs. Only one candidate vQTL region overlapped between the two methods, on SSC7, which also contained the most significant SNP. Within this vQTL region, two candidate genes were identified, *ADGRF1*, which is involved in neurodevelopment of the brain, and *ADGRF5*, which is involved in the function of the respiratory system and in vascularization. The correlation between estimated breeding values based on the two methods was 0.86. Three-fold cross-validation indicated that DHGLM yielded EBV that were much more accurate and had better prediction of missing observations than LnVar.

**Conclusions:**

The results indicated that the LnVar and DHGLM methods resulted in genetically different traits. Based on their validation, we recommend the use of DHGLM over the simpler method of log-transformed variance of residuals. These conclusions can be useful for future studies on the evaluation of the variability of any trait in any species.

**Supplementary Information:**

The online version contains supplementary material available at 10.1186/s12711-021-00692-5.

## Background

Living organisms are under the constant influence of unpredictable changes in the environment. Thus, in ecology as well as in animal and plant breeding, genes that can buffer the negative effects of unpredictable (e.g., diseases) or difficult to avoid (e.g., temperature changes) environmental factors are highly desirable [1]. It is assumed that such genes can control the variation of a trait (at either the population or the individual level) and maintain it at an optimal level [2]. One of the most well-studied genes that is involved in buffering the effects of genetic and environmental factors is the *heat-shock protein 90* (*Hsp90*) gene. In *Drosophila* and *Arabidopsis*, *Hsp90* is described as a stabilizer of developmental and morphological traits [3–5]. This suggests that traditionally applied methods that focus on the genetic control of the mean of traits could be extended by also accounting for the variability around that mean. This is possible since it has been observed that not only the mean of the trait is under genetic control, but also the variation around the mean, which is described in the literature as “variance heterogeneity” or “phenotypic variability”.

Phenotypic variability can be assessed by studying the heterogeneity of residual variance across observations [6]. Empirical evidence that residual variance has a genetic component has been reported for different traits in many animal species [7–15] and in humans [16, 17]. Recently, residual variance has even been linked with filling part of the gap of “missing heritability” in genome-wide association studies (GWAS) in humans [17]. One of the most common methods used to obtain variability phenotype is the double hierarchical generalized linear model (DHGLM) [18]. This rather complex method requires substantial computation time. Thus, to verify the need to use DHGLM, it was compared with simpler approaches: log-transformed variance of a trait [14], log-transformed squared estimated residuals [19], and log-transformed variance of residuals (LnVar) [15]. However, only Sell-Kubiak et al. [14] and Iung et al. [19] reported comprehensive comparative studies. Thus, the extended evaluation would also be needed for LnVar and DHGLM. Furthermore, many studies have reported quantitative trait loci (QTL) that are associated with phenotypic variability, the so-called variance QTL (vQTL) [20]. Detection of vQTL in a population can indicate the presence of an unmodeled interaction associated with the locus [1, 2, 20, 21] or the presence of QTL that directly control the variance of a trait [22, 23]. An overview of selected vQTL that have been detected to date is in Table 1. Still, so far no study has compared the genomic background of variability phenotypes for the same trait obtained with different methods.

**Table 1:** Selected examples of vQTL that affect the variability of quantitative traits across species

Authors	Species	Trait
Mackay and Lyman [53]	*Drosophila melanogaster*	Bristle number
Ordas et al. [54]	Maize	Days to flowering, ear height, and tassel length
Paré et al. [55]	Human	Levels of inflammatory biomarkers
Ayroles et al. [56]	*Drosophila melanogaster*	Locomotor handedness
Perry et al*.* [57]	F2 cross of the genetically hypercalciuric *Rattus norvegicus* with normocalciuric Wistar-Kyoto	Urinary calcium levels
Wolc et al. [58]	Laying hens	Egg weight
Jimenez-Gomez et al. [59]; Shen et al*.* [2]	*Arabidopsis thaliana*	Flowering time
Yang et al. [16]	Human	Body mass index (BMI)
Mulder et al*.* [60]	Dairy cows	Somatic cell score (SCS)
Nelson et al*.* [20]	*S. cerevisiae* strains	Expression traits in different treatments
Sell-Kubiak et al. [25]	Pigs	Litter size
Ek et al. [61]	Human	Variation in DNA methylation levels
Wang et al. [62]; Wang et al. [63]	Pigs	Birth weight
Iung et al. [64]	Nellore cattle	Yearling weight
Hussain et al. [65]	Bread wheat	Cadmium levels

In the current study, we continue to focus on the variability of litter size in a Large White population. Litter size is a trait of high economic relevance for pig breeding and has been under intense selection in the past decades. Many reports have shown that litter size has increased from an average of 11.7 live piglets in 2000 to 17.5 in 2019 [24] and this increase has also led to an increase in its variability, which is due to the positive genetic correlation between the mean litter size and its variability [25]. Although litter size might be one of the most studied traits in pigs in terms of genetics, with more than 255 associated single nucleotide polymorphisms (SNPs) reported between 2011 and 2021 [26], to our knowledge, only the Sell-Kubiak et al. [25] study has detected vQTL for litter size in pigs. One of the most promising candidate genes for the variability of litter size is *HSPCB*, which is better known as the aforementioned *Hsp90* [27]. Although our previous study [25] reported the first SNPs associated with phenotypic variability in litter size in pigs, a follow-up study is necessary to confirm more precisely the genomic regions that affect variability in litter size, especially because now we have access to high-density SNP-chip data (660 K instead of 60 K) and the number of genotyped animals has increased exponentially since the publication of our previous paper.

Here, we compare two methods to estimate the phenotypic variability of litter size and to evaluate its genetic background and detect new genomic regions associated with it, by performing a GWAS using genotypes from a high-density SNP-chip. Thus, our study has two main aims: (1) to compare two approaches for obtaining variability phenotypes for litter size, and for analyzing their genetic background, i.e. a simpler log-transformed variance of residuals (LnVar) and a more complex double hierarchical generalized linear model (DHGLM); and (2) to perform a single-SNP GWAS to detect novel genomic regions associated with litter size variability using high-density SNP-chip data on ~ 12,000 Large White pigs. The data used in this study is an updated version of the litter size records and genotypes used in the previous GWAS performed by Sell-Kubiak et al. [25], where only DHGLM was used to obtain variability phenotypes.

## Methods

### Phenotypes

**Fig. 1:**
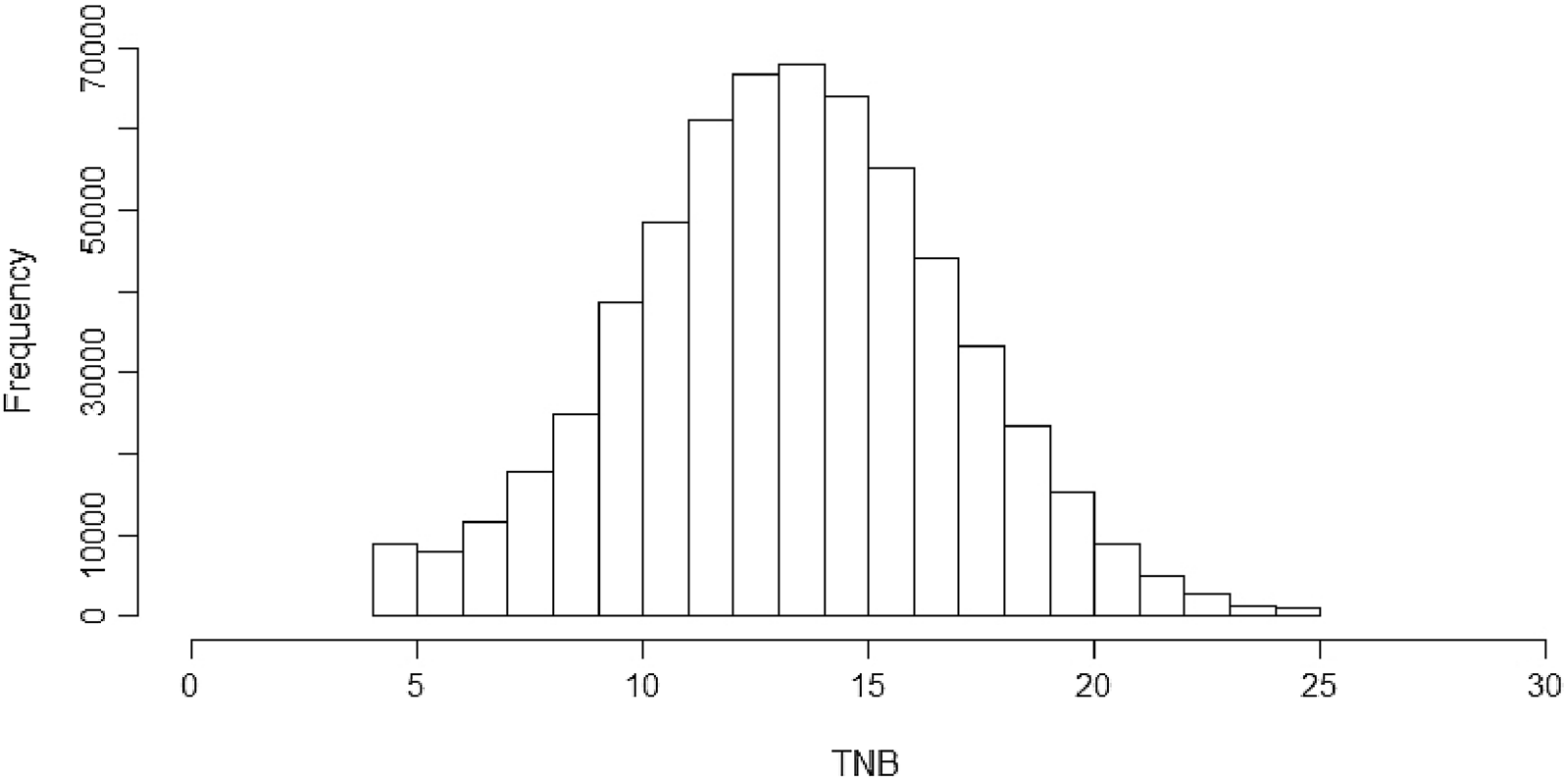
Histogram of the distribution of litter size (*TNB* total number born)

The phenotypic data on Large White pigs used in this study were collected on multiplication farms of Topigs Norsvin (Vught, the Netherlands) between March 1982 and January 2019. In total, litter size records (total number born, TNB) for over 640,000 litters were available before data editing. Records were removed if TNB was equal to 3 or less, or if only one record per sow was available. Parities 10 + were treated as parity 10 and litters with a TNB larger than 25 were recoded to 25. This allowed us to keep such records in the analysis, rather than removing the most extreme values. After data editing, 607,553 litter records from 121,088 sows were available for further analysis. The average TNB was 13.76 (± 3.64) and the distribution of TNB is presented in Fig. 1. The pedigree contained 168,230 records and was on average five generations deep.

### Estimation and analysis of variability phenotypes using the log-transformed variance of residuals

Variability phenotype was obtained as the log-transformed variance of residuals (LnVar) of TNB in two steps. First, to obtain estimates of variance components, the TNB data were analyzed with the following traditional animal model, as previously described and tested by Dobrzański et al. [28], using the ASReml 3.0 package [29]:

1 $$\mathbf{y}=\mathbf{X}\mathbf{b}+\mathbf{Z}\mathbf{a}+\mathbf{U}\mathbf{p}\mathbf{e}+\mathbf{e},$$

where $\mathbf{y}$ is a vector of phenotypes for TNB; $\mathbf{X}$, $\mathbf{Z}$, and $\mathbf{U}$ are incidence matrices relating phenotypes to effects; $\mathbf{b}$ is a vector of the fixed effects of herd-year-season and parity; $\mathbf{a}$ is a vector of additive genetic effects, with $\mathbf{a}{\sim}{\text{N}}\left({\mathbf{0}},\mathbf{A}{\sigma }_{{a}}^{2}\right)$, where $\mathbf{A}$ is the augmented numerator relationship matrix and ${\sigma }_{{a}}^{2}$ is the additive genetic variance; $\mathbf{p}\mathbf{e}$ is a vector of permanent environmental sow effects, which accounts for repeated observations per sow, with $\mathbf{p}\mathbf{e}{\sim }{\text{N}}\left({\mathbf{0}},{\mathbf{I}}_{\mathrm{pe}}{\sigma }_{{pe}}^{2}\right),$ where ${\mathbf{I}}_{\mathrm{pe}}$ is the appropriate identity matrix and ${\sigma }_{{pe}}^{2}$ is the permanent environmental sow variance; and $\mathbf{e}$ is a vector of residuals, with $\mathbf{e}{\sim}{\text{N}}\left({\mathbf{0}},{\mathbf{I}}_{\mathrm{e}}{\sigma }_{{e}}^{2}\right)$, where ${\mathbf{I}}_{\mathrm{e}}$ is the appropriate identity matrix and ${\sigma }_{{e}}^{2}$ is the residual variance.

In the second step, estimates of the residuals from the first step were used to estimate the phenotype for the log of variability of TNB (LnVarTNB) for each sow as the within-individual variance of residuals and log-transforming the result. Log transformation was used to normalize the distribution of the variability phenotypes and to assume an exponential model for the environmental variance [8, 30–32], which in general is described as: ${y}_{i}=\mu +u+\mathrm{exp}\left(\frac{1}{2}\left(\eta +v\right)\right){\epsilon }_{j}\,{\text{for}}\,j=1,\dots n$, where ${y}_{i}$ are the phenotypic observations for LnVarTNB, $\mu$ is the population mean, $\eta$ is the log environmental variance mean; $u$ and $v$ are the genetic values of the mean and environmental variance, respectively, following $$\left[\begin{array}{c}u\\ v\end{array}\right]{\sim}\text{ N}\left({\mathbf{0}},\left[\begin{array}{cc}{\sigma }_{{u}}^{2}& {\sigma }_{{u,v}}\\ {\sigma }_{{u,v}}& {\sigma }_{{v}}^{2}\end{array}\right]\right)$$, where ${\sigma }_{{u}}^{2}$ and ${\sigma }_{{v}}^{2}$ are the genetic variances and ${\sigma }_{{u,v}}$ is the covariance between the genetic effects; and ${\epsilon }_{j}$ refers to independent, identically and normally distributed variables, that are independent from $u$ and $v$.

The variability phenotypes from LnVar were analyzed with the following model, as previously used by Berghof et al. [15] and Dobrzański et al. [28]:

2 $${\mathbf{y}}_{\mathbf{v}\mathbf{a}\mathbf{r}}={\mathbf{X}}_{\mathbf{v}\mathbf{a}\mathbf{r}}{\mathbf{b}}_{\mathbf{v}\mathbf{a}\mathbf{r}}+{\mathbf{Z}}_{\mathbf{v}\mathbf{a}\mathbf{r}}{\mathbf{a}}_{\mathbf{v}\mathbf{a}\mathbf{r}}+{\mathbf{e}}_{\mathbf{v}\mathbf{a}\mathbf{r}},$$

where ${\mathbf{y}}_{\mathbf{v}\mathbf{a}\mathbf{r}}$ is a vector of LnVarTNB phenotypes; ${\mathbf{X}}_{\mathbf{v}\mathbf{a}\mathbf{r}}$ and ${\mathbf{Z}}_{\mathbf{v}\mathbf{a}\mathbf{r}}$ are incidence matrices relating phenotypes to model effects; ${\mathbf{b}}_{\mathbf{v}\mathbf{a}\mathbf{r}}$ is a vector of the fixed effects of farm-year-season of the first farrowing; ${\mathbf{a}}_{\mathbf{v}\mathbf{a}\mathbf{r}}$ is a vector of additive genetic effects, with ${\mathbf{a}}_{\mathbf{v}\mathbf{a}\mathbf{r}}{\sim}\text{ N}\left({\mathbf{0}},\mathbf{A}{\sigma }_{\text{avar}}^{2}\right)$, where ${\sigma }_{\text{avar}}^{2}$ is the additive genetic variance; and ${\mathbf{e}}_{\mathbf{v}\mathbf{a}\mathbf{r}}$ is a vector of residuals, with ${\mathbf{e}}_{\mathbf{v}\mathbf{a}\mathbf{r}}{\sim}\text{ N}\left({\mathbf{0}},{\mathbf{I}}_{\mathrm{evar}}{\sigma }_{\text{evar}}^{2}\right)$, where ${\mathbf{I}}_{\mathrm{evar}}$ is the appropriate incidence matrix and ${\sigma }_{\text{evar}}^{2}$ is the residual variance. To account for differences in residual variance due to the varying numbers of TNB records available per sow, we estimated a unique residual variance for each of the nine groups of sows based on numbers of records (Group 1: 19,670 sows with two litters; Group 2: 18,173 sows with three litters; Group 3: 17,601 sows with four litters; Group 4: 16,868 sows with five litters; Group 5: 15,661 sows with six litters; Group 6: 13,365 sows with seven litters; Group 7: 9516 sows with eight litters; Group 8: 5581 sows with nine litters; and Group 9: 4188 sows with ten or more litters).

### Estimation and analysis of variability using a double hierarchical generalized linear model

We also analyzed the variability of TNB with a double hierarchical generalized linear model (DHGLM) [18, 33], which also allows estimation of variance components for residual variance in ASReml 3.0. The method is based on a bivariate model that requires several iterations until convergence and was used in an earlier study to obtain variability phenotypes for a GWAS for litter size variability [25]. The applied DHGLM was:

3 $$\left[\begin{array}{c}\mathbf{y}\\ {\varvec{\uppsi}}\end{array}\right]=\left[\begin{array}{cc}\mathbf{X}& {\mathbf{0}}\\ {\mathbf{0}}& {\mathbf{X}}_{\mathbf{v}}\end{array}\right]\left[\begin{array}{c}\mathbf{b}\\ {\mathbf{b}}_{\mathbf{v}}\end{array}\right]+\left[\begin{array}{cc}\mathbf{Z}& {\mathbf{0}}\\ {\mathbf{0}}& {\mathbf{Z}}_{\mathbf{v}}\end{array}\right]\left[\begin{array}{c}\mathbf{a}\\ {\mathbf{a}}_{\mathbf{v}}\end{array}\right]+\left[\begin{array}{cc}\mathbf{U}& {\mathbf{0}}\\ {\mathbf{0}}& {\mathbf{U}}_{\mathbf{v}}\end{array}\right]\left[\begin{array}{c}\mathbf{p}\mathbf{e}\\ \mathbf{p}{\mathbf{e}}_{\mathbf{v}}\end{array}\right]+\left[\begin{array}{c}\mathbf{e}\\ {\mathbf{e}}_{\mathbf{v}}\end{array}\right],$$

where $\mathbf{y}$ is the vector of phenotypes TNB and ${\varvec{\uppsi}}$ is the vector of response variables for the variance part of the DHGLM; the residuals $\mathbf{e}$ and ${\mathbf{e}}_{\mathbf{v}}$ are assumed to be independent and normally distributed but with heterogeneous variances across phenotypes; $\mathbf{b}$ and ${\mathbf{b}}_{\mathbf{v}}$ are vectors of the fixed effects of parity of the sow and farm-year-season of the farrowing for $\mathbf{y}$ and ${\varvec{\uppsi}}$, respectively; $\mathbf{a}$ and ${\mathbf{a}}_{\mathbf{v}}$ are vectors of random additive genetic effects for $\mathbf{y}$ and ${\varvec{\uppsi}}$, with $$\left[\begin{array}{c}{\mathbf{a}}\\ {\mathbf{a}}_{\mathbf{v}}\end{array}\right]{\sim}\text{ N}\left({\mathbf{0}},\left[\begin{array}{cc}{\sigma }_{\text{a}}^{2}& {\sigma }_{\text{a,av}}\\ {\sigma }_{\text{a,av}}& {\sigma }_{\text{av}}^{2}\end{array}\right]\otimes \mathbf{A}\right)$$, where ${\sigma }_{\text{av}}^{2}$ is the additive genetic variance, ${\sigma }_{\text{a,av}}$ is the covariance between the genetic effects and $\otimes$ is the Kronecker product; $\mathbf{p}\mathbf{e}$ and $\mathbf{p}{\mathbf{e}}_{\mathbf{v}}$ are vectors of random non-genetic permanent sow effects for $\mathbf{y}$ and ${\varvec{\uppsi}}$·, with $$\left[\begin{array}{c}\mathbf{p}\mathbf{e}\\ \mathbf{p}{\mathbf{e}}_{\mathrm{v}}\end{array}\right]{\sim}\text{ N}\left({\mathbf{0}},\left[\begin{array}{cc}{\sigma }_{\text{pe}}^{2}& {\sigma }_{\text{pe,pev}}\\ {\sigma }_{\text{pe,pev}}& {\sigma }_{\text{pev}}^{2}\end{array}\right]\otimes \mathbf{I}\right)$$, where ${\sigma }_{\mathrm{pev}}^{2}$ is the permanent sow variance, ${\sigma }_{\text{pe,pev}}$ is the covariance between the permanent sow effects; and $\mathbf{e}$ and ${\mathbf{e}}_{\mathbf{v}}$ are vectors of residuals, with $$\left[\begin{array}{c}\mathbf{e}\\ {\mathbf{e}}_{\mathrm{v}}\end{array}\right]{\sim}\text{ N}\left(\begin{array}{c}{\mathbf{0}}\\ {\mathbf{0}}\end{array},\left[\begin{array}{cc}{\mathbf{W}}^{-1}{\sigma }_{\mathrm{e}}^{2}& 0\\ 0& {\mathbf{W}}_{\mathrm{v}}^{-1}{\sigma }_{\text{ev}}^{2}\end{array}\right]\right)$$, where $\mathbf{W}={\text{diag}}\left({\text{exp}}{\left(\stackrel{\wedge }{\uppsi }\right)}^{-1}\right)$ and ${\mathbf{W}}_{\mathrm{v}}={\text{diag}}\left(\frac{1-h}{2}\right)$ are expected reciprocals of the residual variance from the previous iteration, and ${\sigma }_{\mathrm{e}}^{2}$ and ${\sigma }_{\text{ev}}^{2}$ are scaling variances and are expected to be equal to 1 [31]. The predicted residual variance for each TNB phenotype ${\text{exp}}\left(\stackrel{\wedge }{\uppsi }\right)$ is based on the estimated fixed and random effects for ${\varvec{\uppsi}}$ in the previous iteration of the algorithm. This methodology produced the second variability phenotype—varTNB and estimated its variance components.

Note: to fully evaluate the genetic variation within litter size variability based on LnVar and DHGLM, the genetic coefficient of variation on the standard deviation (SD) level (${\mathrm{GCV}}_{\mathrm{SDe}}$) was used, which can be approximated as: ${\mathrm{GCV}}_{\mathrm{SDe}}=\frac{{\upsigma }_{\mathrm{addv}}\left({\sigma }_{\mathrm{e}}\right)}{\overline{{\sigma }_{\mathrm{e}}}}\approx \frac{1}{2}{\sigma }_{\mathrm{avar}}$ or $\approx \frac{1}{2}{\sigma }_{\mathrm{av}}$, where ${\upsigma }_{\mathrm{addv}}$ is the genetic SD in residual variance.

### Comparison of the LnVar and DHGLM

The two methods used to estimate variability phenotypes for litter size, i.e. LnVar and DHGLM, were evaluated by:

1. Comparing the estimates of variance components and the estimated breeding values (EBV) of LnVarTNB and varTNB and their theoretical accuracies which were estimated based on the following equation: ${r}_{A,I}=\sqrt{1-\frac{{s}_{i}}{{\sigma }_{\mathrm{add}}^{2}}}$, where ${s}_{i}$ is the standard error for the EBV of the $i$th individual and ${\sigma }_{\mathrm{add}}^{2}$ is the additive genetic variance for the trait.
2. Three-fold cross-validation used to predict EBV for LnVarTNB and varTNB. For this, we selected paternal families with at least three half-sisters with litter size records. Then, phenotypes for one of the paternal half-sisters were set to missing, which resulted in removing 3650 sows and their records in three subsets of data. This cross-validation imitates a situation where a breeding program aims at predicting the phenotype of a new-born sow that already has paternal half-sisters with litter size records. The predicted EBV were then correlated with the log-transformed variance of litter size per sow. A similar validation approach was described by Sell-Kubiak et al. [14].

### SNP genotypes for GWAS

In total, 12,232 genotyped sows (N = 11,451) and boars (N = 781) were available for the GWAS. Genotyping was performed at GeneSeek (Lincoln, NE, USA) using three medium-density SNP chips. Most animals (N = 7079) were genotyped using the (Illumina) GeneSeek custom 50K SNP chip (Lincoln, NE, USA), while 3276 and 1877 animals were genotyped using the (Illumina) GeneSeek custom 80K SNP chip (Lincoln, NE, USA) and the Illumina Porcine SNP60 Beadchip (Illumina, San Diego, CA, USA), respectively. In total, 499 animals that had already been genotyped with the medium-density chips were also genotyped with the Axiom porcine 660K array from Affymetrix (Affymetrix Inc., Santa Clara, CA, USA) at GeneSeek (Lincoln, NE, USA). These animals were the sires with the largest number of offspring in the genotyped dataset.

Quality control of the genotype data included exclusion of SNPs with a GenCall score < 0.15 (Illumina Inc., 2005), a call rate < 0.95, and a minor allele frequency < 0.01, and of the SNPs that deviated strongly from the Hardy–Weinberg equilibrium (χ^2^ > 600), that were located on sex chromosomes, and that were unmapped. The positions of the SNPs were based on the Sscrofa11.1 assembly of the reference genome. Since all animals had a frequency of missing genotypes ≤ 0.05, none were removed based on that criterion.

After quality control, missing genotypes of the animals genotyped with the (Illumina) GeneSeek custom 50K SNP chip were imputed within the population using the Fimpute v2.2 software [34], while animals genotyped with the other two chips had their genotypes imputed to the set of SNPs on the (Illumina) GeneSeek custom 50K SNP chip that passed the quality control. After quality control and imputation, genotypes on 50,717 SNPs were available and were used in imputation to the 660K genotypes using Fimpute v2.2 [34]. After quality control and imputation, genotypes on 526,505 SNPs were available for the GWAS.

### Genome-wide association study

For use in GWAS, the EBV for variability obtained with the two methods were deregressed using the methodology of Garrick et al*.* [35]. Deregression of EBV avoids double-counting of parental information due to various information sources and complex family structures in a population, and provides phenotypes for boars that do not have own litter size records. This also allows more genotyped animals to be included in the GWAS and thus increase its power to detect QTL.

A single-SNP GWAS was performed using the imputed 660K genotypes and applying the following linear animal model in the GCTA software [36, 37]:

4 $${y}_{k}^{*}= \mu + {\mathrm{X}}^{*}\widehat{\upbeta } + {u}_{k}^{*} + {e}_{k}^{*},$$

where ${y}_{k}^{*}$ is the deregressed EBV of the $k$th animal for LnVarTNB or varTNB; $\mu$ is the average of the deregressed EBV; ${\mathrm{X}}^{*}$ is the genotype (0, 1, 2) of the $k$th animal for the evaluated SNP; $\widehat{\upbeta }$ is the unknown allele substitution effect of the evaluated SNP; ${u}_{k}^{*}$ is the random additive genetic effect, assumed to be distributed as $\sim N({\mathbf{0}},\mathbf{G}{\sigma }_{\mathrm{a}}^{2*})$, which accounts for the (co)variances between animals though the formation of a genomic numerator relationship matrix ($\mathbf{G}$), constructed using the imputed 660 K genotypes, ${\sigma }_{\mathrm{a}}^{2*}$ is the additive genetic variance; and ${e}_{k}^{*}$ is the random residual effect, which was assumed to be distributed as $\sim N({\mathbf{0}},\mathbf{I}{\sigma }_{\mathrm{e}}^{2*})$.

The genetic variance explained by a SNP (${\sigma }_{snp}^{2}=2pq{\widehat{\upbeta }}^{2}$) was estimated based on the allele frequencies ($p$ and $q$) and the estimated allele substitution effect ($\widehat{\upbeta }$). The proportion of phenotypic variance explained by the SNP was defined as ${\sigma }_{snp}^{2}/{\sigma }_{P}^{2}$, where ${\sigma }_{P}^{2}$ is the total phenotypic variance (sum of the additive genetic and residual variances), which was estimated based on the model described above without a SNP effect. Significant SNPs were declared based on a p-value < 10^–6^, while suggestive SNPs were declared based on a p-value < 10^–4^ and linkage disequilibrium (LD) with the significant SNP. Each region with the significant and suggestive SNPs was defined as a separate vQTL region.

Following the GWAS based on the imputed 660 K data, we investigated the linkage disequilibrium (LD) in the QTL region on SSC7 that overlapped between the two variability phenotypes. LD, as measured by r^2^, was calculated between SNPs using the Plink 1.9 software [38].

### Search for candidate genes

Based on the GWAS results, we used the location of the significant SNPs to search for candidate genes with the software BIOMART available in Ensembl Sscrofa 11.1 [39] by entering the position of a SNP and ± 50 kb if the SNP was not located within a known gene. Furthermore, the PigQTL database of the Animal Genome project [26] was used to find previously detected associations and QTL related to pig reproduction within the most promising regions. This was done by entering the start and end position of the identified QTL regions for litter size variability.

## Results

### Comparison of the variability phenotypes

Estimates of the variance components and heritability for total number born (TNB) and its variability are in Table 2. The variance components obtained for the variability of litter size differed between the two estimation methods (LnVar and DHGLM), which resulted in differences between heritability estimates. The genetic coefficient of variation on the SD level (${\mathrm{GCV}}_{\mathrm{SDe}}$) indicated a possible change of 9.1 and 9.6% per generation in the genetic SD of LnVarTNB and of varTNB, respectively. Table 3 shows estimates of the correlations between random effects in the DHGLM, with a correlation of 0.43 between additive effects and a correlation of − 0.87 between permanent sow effects.

**Table 2:** Estimates of variance components, heritability, and the genetic coefficient of variation on the standard deviation level (${\text{G}\text{C}\text{V}}_{\text{S}\text{D}\text{e}}$)

Estimate	TNB	LnVarTNB	varTNB
Permanent sow variance	0.86 (0.01)	–	0.15 (0.003)
Additive genetic variance	1.33 (0.2)	0.033 (0.001)	0.037 (0.002)
Residual variance	7.67 (0.8)	1.33 (0.4)	0.84
Heritability	0.135 (0.02)	0.024 (0.002)^a^	0.036^a^
${\mathrm{GCV}}_{\mathrm{SDe}}$^b^	–	0.091	0.096

TNB: litter size as total number born

LnVarTNB: phenotypic variability of litter size estimated by the log-transformed variance of residuals

varTNB: phenotypic variability estimated with double hierarchical generalized linear model measured in 121,088 sows from the Large White pig population

^a^Heritability is a measure of the reliability of EBV for LnVarTNB and varTNB; it does not reflect the magnitude of the genetic variance in varTNB

^b^Measure of the genetic variation in residual variance ${\mathrm{GCV}}_{\mathrm{SDe}}=\frac{{\upsigma }_{\mathrm{a}ddv}\left({\upsigma }_{\mathrm{e}}\right)}{\overline{{\upsigma }_{\mathrm{e}}}}\approx \frac{1}{2}{\upsigma }_{\mathrm{avar}} \,{\text{or} }\,\approx \frac{1}{2}{\upsigma }_{\mathrm{av}}$

**Table 3:** Estimates of correlations^a^ (with SE) between the random effects on litter size and its variance

Effect	Correlation
Additive genetic	0.43 (0.03)
Permanent sow	− 0.87 (0.02)

^a^Obtained with double hierarchical GLM

The correlation between the EBV obtained with the two methods was 0.86, which indicates some reranking of the animals. In the validation, we also compared the EBV from the two methods with those obtained for the variability of litter size by Sell-Kubiak et al. [25], which will be referred to as varTNB_2015. Estimates of the correlation of the EBV for varTNB_2015 with those for LnVarTNB and varTNB were 0.73 and 0.83, respectively. These results suggest that the variability phenotypes obtained with the two methods are not as similar as expected.

**Fig. 2:**
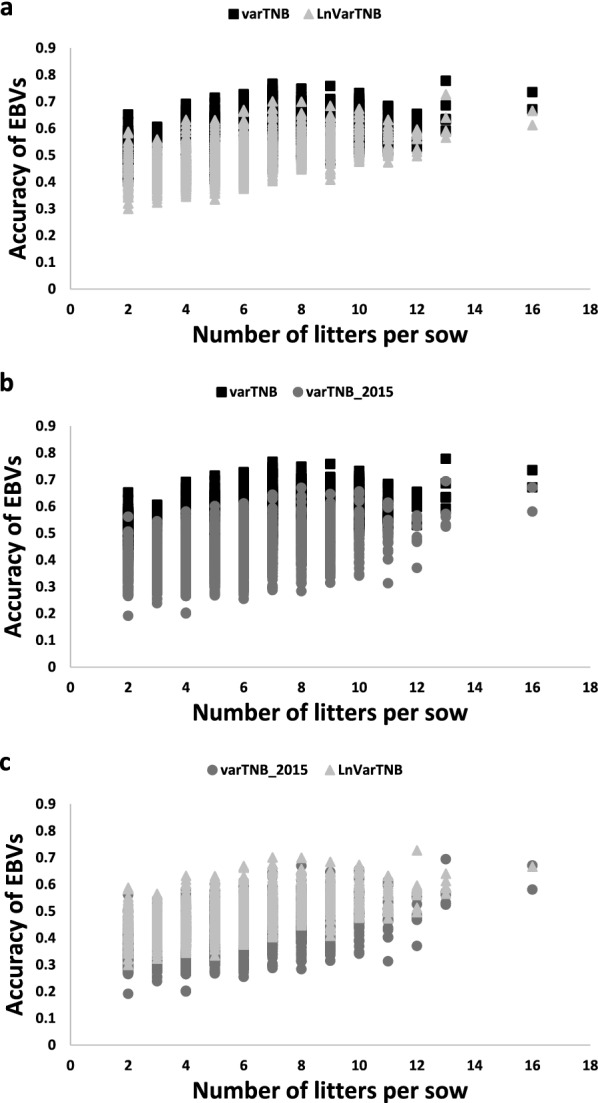
Theoretical accuracies of EBV for litter size variability in Large White sows for two methods: log-transformed variance of residuals from multiple observations per sow (LnVarTNB) and residual variance of individual litter size obtained with a double hierarchical GLM (varTNB; or varTNB_2015 based on Sell-Kubiak et al. [25]). **a** LnVarTNB vs. varTNB, **b** varTNB vs. varTNB_2015, **c** LnVarTNB vs. varTNB_2015

The two methods were also compared by evaluating the theoretical accuracies of the EBV and by cross-validation. The theoretical accuracies presented in Fig. 2 indicate that the highest accuracy was reached for varTNB, while LnVarTNB and varTNB_2015 resulted in very similar accuracies, although the older dataset contained fewer records per sow. The results of the three-fold cross-validation indicated that the EBV from DHGLM had a significantly (P = 0.038 based on a t-test) higher precision than the EBV based on LnVar (Table 4).

**Table 4:** Correlations between log-transformed variance of litter size and EBV for LnVar^c^ and DHGLM^c^ obtained from three-fold cross-validation^a^

Validation run	Correlations of ln(var(TNB))^b^ with	
	EBV from LnVar^c^	EBV from DHGLM^c^
1	0.47	0.55
2	0.42	0.49
3	0.40	0.44
Average	0.43	0.49

^a^Three-fold cross-validation was performed to compare the two methods used to obtain phenotypic variability of litter size: log-transformed variance of residuals (LnVar) and double hierarchical GLM (DHGLM)

^b^Log-transformed variance of litter size per sow

^c^Estimated in each run for 3650 sows with their records set to missing

### Significant SNPs and candidate genes

**Fig. 3:**
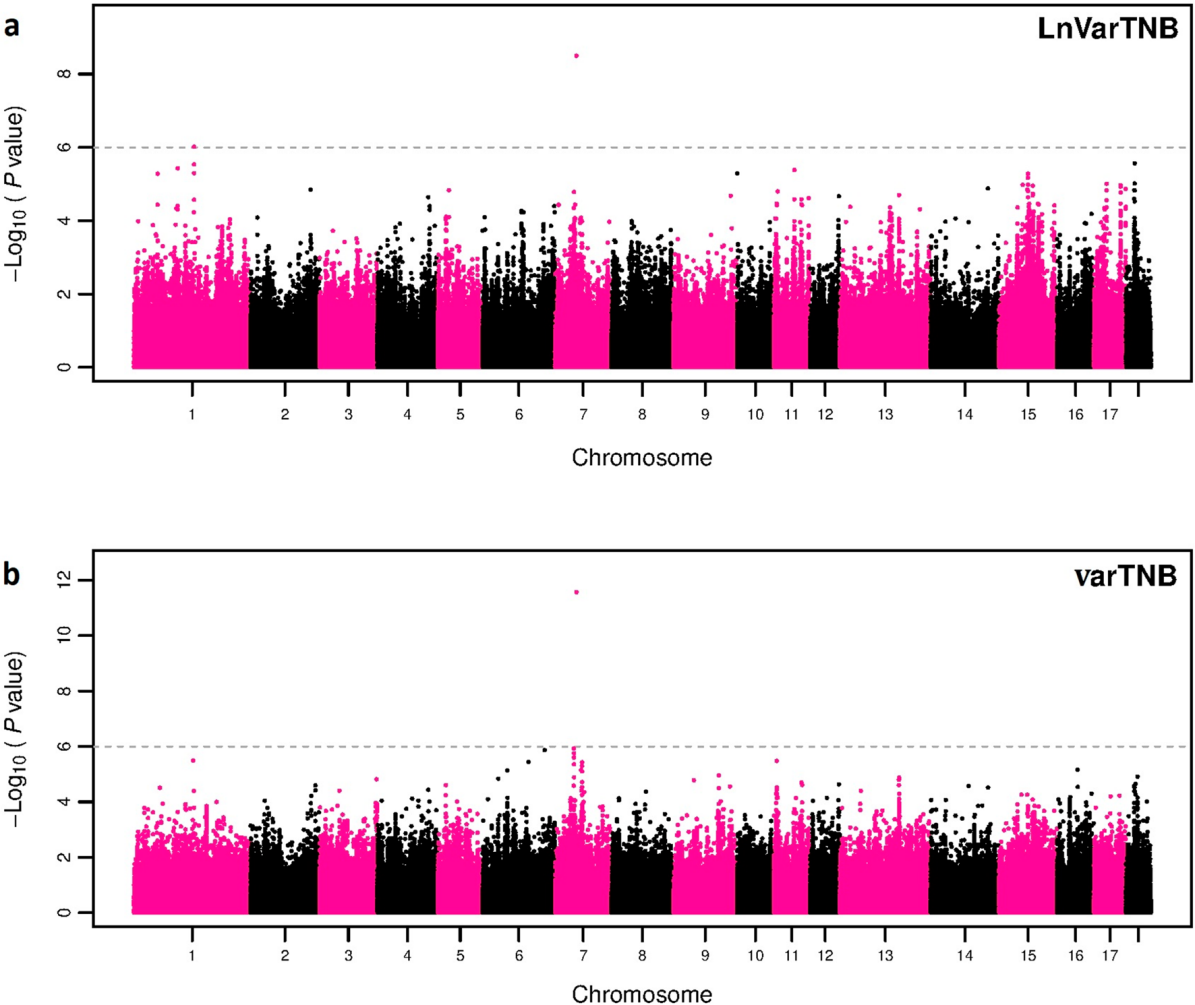
Genome-wide association for phenotypic variability of litter size in Large White pigs based on **a** deregressed EBV of log-transformed residuals (LnVarTNB) for 11,230 genotyped purebred sows and boars, and **b** a double hierarchical generalized linear model (varTNB) for 12,232 genotyped purebred sows and boars. The dashed grey line on the figure indicates the significance threshold − log_10_(P-value) ≤ 6

**Fig. 4:**
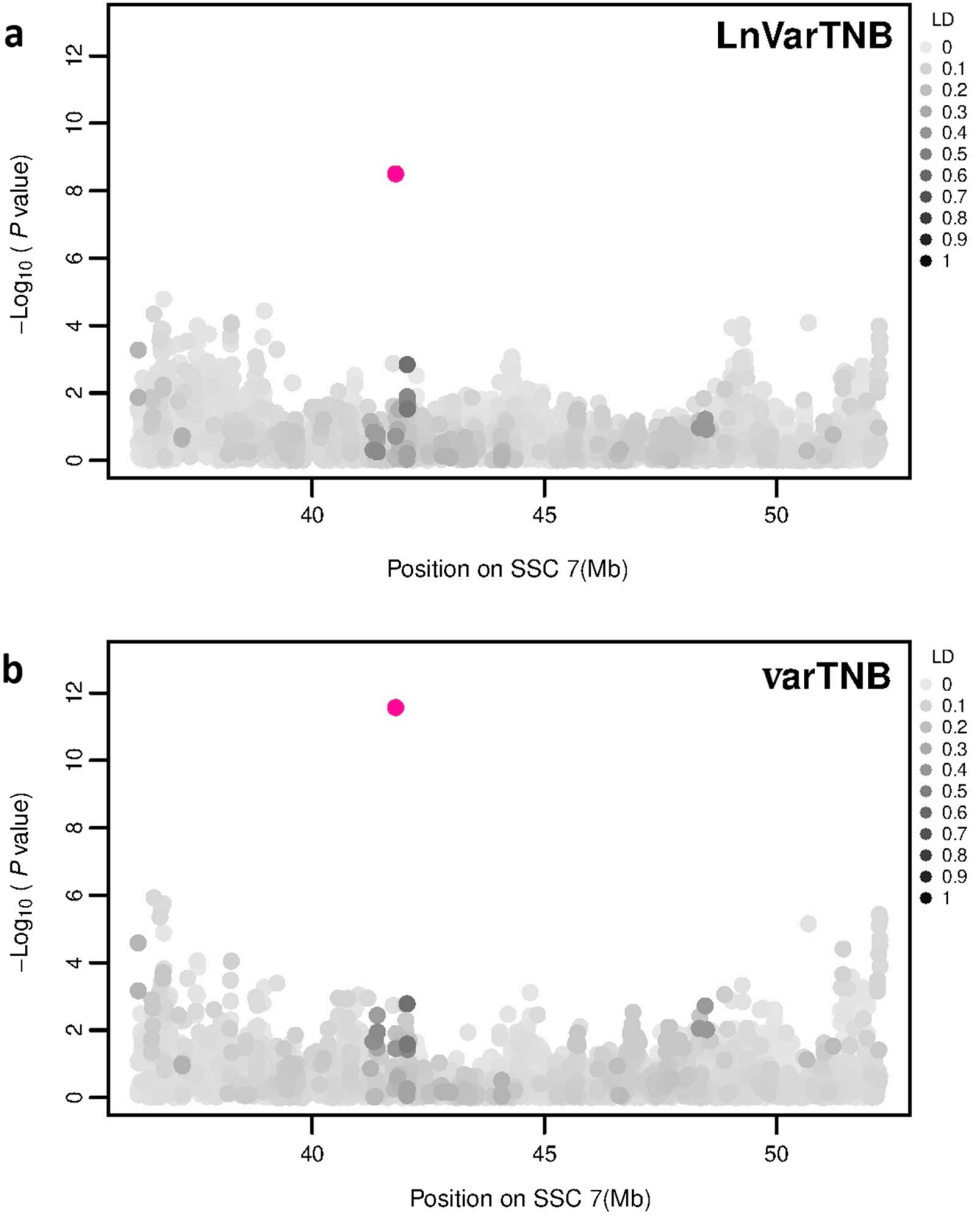
Manhattan plots for the GWAS of phenotypic variability of litter size based on **a** LnVarTNB, and **b** varTNB. The y-axis shows the − log_10_(p-values) of single SNP association with LnVarTNB or varTNB in Large White pigs, and the x-axis shows the physical position of the SNP in the SSC7 vQTL region. Linkage disequilibrium (LD) is given on a scale of 0–1 as a measure of the pairwise correlation between the most significant SNP (pink dot) and all other SNPs

Figures 3 and 4 present the GWAS results of this study and Tables 5 and 6 show the significant and suggestive SNPs for each identified vQTL region for LnVarTNB and varTNB, respectively. The estimates of the genetic variance obtained in the GWAS were 0.012 for LnVarTNB and 0.014 for varTNB. The inflation factors (lambda) for both variability phenotypes are in Additional file 1: Fig. S1. Only one identified vQTL region overlapped between LnVarTNB and varTNB, i.e. a region on SSC7, which contained the most significant SNP detected for both variability traits, AX-116689108. This SNP had a low minor allele frequency (MAF = 0.01) with only three animals being homozygous for the least frequent genotype and 253 animals being heterozygous. The least frequent allele was associated with greater litter size variability. This low MAF could be explained by the selection history of the population, with strong selection for increased litter size over the last decades, because greater litter size variation could reduce the average litter size.

**Table 5:** Most significant *(in italics)* and suggestive SNPs in identified vQTL for litter size variability^a^

SSC	Name	Position (Mb)	MAF	SNP effect (with SE)	Genetic. variance explained	Phenotypic variance explained	− log(p-value)	SNP location	Candidate genes	Previously detected QTL
1	AX-116127462	129.07	0.01	− 0.083 (0.020)	0.013	0.002	4.57	Intron	GANC	None
	AX-116127463	129.08	0.01	− 0.100 (0.021)	0.017	0.003	5.53	Intron	GANC	
	*AX-116127466*	*129.09*	*0.01*	− *0.097 (0.020)*	*0.018*	*0.003*	*6.02*	*Intron*	*GANC*	
	AX-116702728	129.17	0.01	− 0.083 (0.018)	0.015	0.003	5.30	Intron	VPS39	
	AX-116127269	127.69	0.03	− 0.047 (0.012)	0.012	0.002	4.23	Non-coding	None^a^	
7	AX-116317099	36.59	0.47	0.020 (0.005)	0.016	0.003	4.35	Non-coding	NCR2	Birthweight variability [66]; number mummified piglets [67]; age at puberty [68]
	AX-116317159	36.80	0.49	0.019 (0.004)	0.015	0.003	4.78	Non-coding	NRN1	
	AX-116317552	38.26	0.36	0.018 (0.005)	0.013	0.002	4.03	Intron	CUL9	
	AX-116317553	38.26	0.36	0.018 (0.005)	0.013	0.002	4.07	Intron	CUL9	
	AX-116317740	38.97	0.36	− 0.019 (0.005)	0.015	0.003	4.43	Non-coding	None^a^	
	*AX-116689108*	*41.80*	*0.01*	*0.106 (0.018)*	*0.020*	*0.003*	*8.50*	*Non-coding*	*ADGRF1*; *ADGRF5*	
18	AX-116596013	18.07	0.05	− 0.039 (0.009)	0.012	0.002	4.60	Intron	LMBR1	Litter weight [69]; litter size [69]; litter weight of piglets born alive [69]; birthweight [70]
	AX-116596024	18.19	0.09	− 0.030 (0.007)	0.013	0.002	4.83	Non-coding	KLF14; TSGA13; COPG2	
	AX-116596140	18.88	0.04	0.045 (0.011)	0.01	0.002	4.08	Intron	UBE2H	
	AX-116596204	19.30	0.07	− 0.034 (0.008)	0.012	0.002	4.53	Intron	AHCYL2	
	AX-116596216	19.37	0.09	− 0.029 (0.007)	0.011	0.002	5.01	Intron	AHCYL2	
	*AX-116596225*	*19.40*	*0.06*	− *0.041 (0.009)*	*0.015*	*0.003*	*5.56*	*Intron*	*AHCYL2*	
	AX-116596239	19.45	0.05	− 0.039 (0.009)	0.013	0.002	4.82	Intron	SMO	

*SSC* *Sus scrofa* chromosomes; SNPs on each SSC present confirmed candidate QTL region

*MAF* minor allele frequency

^a^Obtained with a log-transformed variance of residuals

^b^Within ± 50 kb

**Table 6:** Most significant *(in italics)* and suggestive SNPs in identified vQTL for litter size variability^a^

SSC	Name	Position (Mb)	MAF	SNP effect (with SE)	Genetic variance explained	Phenotypic variance explained	− log(p-value)	SNP location	Candidate genes	Previously detected QTL
7	AX-116317026	36.25	0.016	0.055 (0.013)	0.007	0.002	4.59	Non-coding	None^a^	Birthweight variability [66]; number mummified piglets [67]; age at puberty [68]
	AX-116317099	36.59	0.470	0.022 (0.004)	0.016	0.004	5.93	Non-coding	NCR2	
	AX-116317136	36.72	0.480	− 0.020 (0.004)	0.014	0.004	5.36	Non-coding	FOXP4	
	AX-116317145	36.74	0.310	− 0.025 (0.005)	0.019	0.005	5.60	Non-coding	FOXP4	
	AX-116317156	36.79	0.310	− 0.026 (0.005)	0.019	0.005	5.76	Exon	FOXP4	
	AX-116317159	36.80	0.490	0.017 (0.004)	0.011	0.003	4.88	Non-coding	MDFI; FOXP4	
	AX-116317341	37.52	0.332	0.017 (0.004)	0.008	0.002	4.06	Intron	TRERF1	
	AX-116317343	37.53	0.332	0.017 (0.004)	0.008	0.002	4.06	Intron	TRERF1	
	AX-116317552	38.26	0.361	0.017 (0.004)	0.009	0.002	4.04	Intron	CUL9	
	AX-116317553	38.26	0.361	0.017 (0.004)	0.009	0.002	4.05	Intron	CUL9	
	*AX-116689108*	*41.80*	*0.010*	*0.110 (0.016)*	*0.018*	*0.005*	*11.57*	*Non-coding*	*ADGRF1; ADGRF5*	
7	AX-116320104	50.68	0.312	0.020 (0.004)	0.012	0.003	5.15	Non-coding	None^a^	Teat number [71]; number mummified piglets [67]
	AX-116320268	51.43	0.248	0.019 (0.005)	0.009	0.002	4.41	Intron	SH3GL3	
	AX-116320489	52.20	0.345	0.017 (0.004)	0.009	0.002	4.25	Non-coding	FSD2; AP3B2	
	AX-116320491	52.20	0.332	0.02 (0.004)	0.012	0.003	5.10	Non-coding	FSD2; AP3B2	
	*AX-116320493*	*52.21*	*0.330*	*0.021 (0.004)*	*0.013*	*0.003*	*5.43*	*Intron*	*AP3B2*	
	AX-116689308	52.22	0.332	0.019 (0.004)	0.011	0.003	4.71	Intron	AP3B2	
	AX-116320498	52.22	0.332	0.019 (0.004)	0.011	0.003	4.53	Intron	AP3B2	
	AX-116320499	52.23	0.339	0.020 (0.004)	0.012	0.003	5.30	Intron	AP3B2	
	AX-116320501	52.23	0.331	0.019 (0.004)	0.011	0.003	4.53	Intron	AP3B2	
	AX-116320502	52.23	0.242	0.019 (0.005)	0.009	0.002	4.52	Intron	AP3B2	
	AX-116321160	57.08	0.069	− 0.028 (0.007)	0.007	0.002	4.00	Non-coding	PSTPIP1; RCN2	
	AX-116321525	58.97	0.057	− 0.035 (0.008)	0.009	0.002	4.35	Intron	ARID3B	
11	AX-116416565	5.71	0.018	− 0.048 (0.012)	0.006	0.002	4.30	Intron	FLT1	Teat number [72]; number mummified piglets [67]; uterine horn length [73]
	AX-116416787	6.87	0.036	0.041 (0.010)	0.008	0.002	4.43	Non-coding	None^4^	
	*AX-116416839*	*7.04*	*0.05*	*0.038 (0.008)*	*0.009*	*0.002*	*5.50*	*Intron*	*KATNAL1*	
	AX-116416845	7.08	0.036	0.041 (0.010)	0.008	0.002	4.49	Intron	KATNAL1	
	AX-116779844	7.30	0.036	0.041 (0.010)	0.008	0.002	4.53	Non-coding	USPL1	
	AX-116416878	7.32	0.038	0.038 (0.010)	0.007	0.002	4.14	Non-coding	USPL1	
	AX-116779846	7.37	0.039	0.037 (0.009)	0.007	0.002	4.28	Non-coding	USPL1; ALOX5AP	
	AX-116851433	7.39	0.039	0.037 (0.009)	0.007	0.002	4.28	Non-coding	USPL1; ALOX5AP	
	AX-116650934	8.77	0.231	0.020 (0.005)	0.01	0.003	4.16	Non-coding	FRY; ZAR1L	

*SSC* *Sus Scrofa* chromosomes; two clusters of SNPs on SSC7 and one on SSC11 present confirmed candidate QTL regions

*MAF* minor allele frequency

^a^Obtained with Double Hierarchical GLM

^b^Within ± 50 kb

We also investigated the LD between AX-116689108 and its surrounding SNPs to check if the mapping of the most significant SNP was correct. This showed that the highest LD was between AX-116689108 and the SNPs in the vQTL region on SSC7, which confirms that the significant SNP is properly mapped.

Additional vQTL regions were identified on SSC1 and SSC18 for LnVarTNB and on SSC7 and SSC11 for varTNB. These GWAS results provide support for considering the variability phenotypes obtained with the two methods as two different traits, since different genomic regions were identified for each of them.

Based on the positions of the significant and suggestive SNPs for the two variability traits, we identified several candidate genes (see Tables 5 and 6) within ± 50 kb from each SNP. However, not all the significant SNPs were located within a known gene region. This was the case for the most significant SNP detected on SSC7 for both traits and for most of the suggestive SNPs for varTNB. Several previously reported QTL related to reproduction traits in pigs were located within the identified vQTL regions (Tables 5 and 6). Interestingly, SNP AX-116317698 at 38.81 Mb on SSC7 in the vQTL region for variability of litter size was previously found to be associated with TNB (see Additional file 1: Fig. S2 and Additional file 2 Table S1).

## Discussion

The main aim of our study was to detect novel genomic regions that are involved in the genetic control of the phenotypic variability of litter size in Large White pigs. In addition to this objective, we also compared two methods to estimate variability phenotypes, i.e. LnVar and DHGLM. This is the second study that reports vQTL for the variability of litter size and the first one to study the genomic differences between variability phenotypes obtained with two methods.

### Comparison of methods

We compared the following two methods for estimating litter size variability: the simple approach of estimating log-transformed variance residuals (LnVar) and a DHGLM. The latter was previously used for analysis of part of the same data as used here by Sell-Kubiak et al. [25]. The LnVar is a simpler method than DHGLM to compute variability phenotypes and requires less computation time, making it more suitable for application in breeding programs. The DHGLM method was described in detail by Berghof et al. [15] and Dobrzański et al. [28]. Dobrzański et al. [28] also tested whether accounting for the “parity curve” in litter size, i.e. a change in average litter size in subsequent parities [40], affected the variability phenotype. This was proposed since extending the model to better fit the phenotypic data should, in theory, yield more accurate estimates of the residual variance. However, no differences in residual variance were found when applying these more complex animal models [28].

The DHGLM is an interesting approach to study the variability of traits because it enables analysis of variation even when only one observation per animal is available [14]. This of course comes with a cost, since more observations per animal increases the heritability of trait variability [41]. It is important since trait variability tends to have a very low heritability in the classical sense [6], whereas in exponential models it should be considered as a measure of the reliability of EBV for variability and as such does not reflect the magnitude of the genetic variance (for this, the genetic coefficient of variation on the SD level is used). Due to its complexity, DHGLM was compared with simpler methods: log-transformed variance of birth weight per litter in pigs [14], log-transformed squared estimated residuals of yearling weight in Nellore beef cattle [19], and log-transformed variance of residual of body weight in layer hens (LnVar) [15]. Sell-Kubiak et al. [14] and Berghof et al. [15] reported high similarity between the variability traits obtained with the compared methods, whereas the Iung et al. [19] indicated that DHGLM provided more accurate estimates. However, only Sell-Kubiak et al. [14] and Iung et al. [19] reported comprehensive comparative studies, whereas Berghof et al. [15] merely presented the genetic correlation between traits. Interestingly, in the study of Sell-Kubiak et al. [14] the correlation between EBV for the studied methods (0.88) was slightly higher than that found between LnVar and DHGLM (0.86) in the current study, whereas in the study of Iung et al. [19] the correlation was only 0.45. In those studies, the accuracy of EBV was always higher for DHGLM than for the simpler method.

Overall, our results indicate that the two methods used to estimate variability phenotypes of litter size (LnVar and DHGLM) do not produce identical traits, based on comparing estimates of variance components, estimated breeding values, and theoretical accuracies of EBV and by a three-fold cross-validation. Therefore, our results do not confirm the study of Berghof et al. [15] that concluded that these two methods yielded the same trait when analyzing body weight variability in layer hens. In addition, the GWAS results of our study reveal that these two traits are controlled by different genomic regions, given that only the region on SSC7 was significant for both traits. This is in line with the presence of some reranking of EBV when comparing LnVar and DHGLM, which after deregression were used as response variable in GWAS. Thus, our results indicate that LnVar and DHGLM yield, to a large extent, genetically different phenotypes of variability. Moreover, we also showed in the three-fold cross-validation that DHGLM provides more accurate EBV. This means that the two methods should not be treated as interchangeable and although DHGLM has a longer computation time and is more difficult to implement in a real-life breeding program, we recommend it over the simpler methods. This is in line with the findings of Sell-Kubiak et al. [14] and Iung et al. [19].

### Significant SNPs and candidate genes

Within each vQTL region for variability of litter size, several candidate genes were identified. We focused only on genes that are located within ± 50 kb from the most significant SNP in each vQTL region. The most significant SNP (AX-116689108) for both variability phenotypes was located on SSC7 within a non-coding region but notably in the middle of a regulatory element (genomic evolutionary rate profiling, GERP) [39]. In our study of 2015 on variability of litter size, which included a much smaller number of animals from the same population (N = 2067), we also identified a SNP on SSC7 that was significantly associated with varTNB_2015 [25]. This SNP was located at 43.76 Mb based on the *Sus scrofa* build 10.2 [25] but at 38.26 Mb in the *Sus scrofa* build 11.1 used in the current study. However, in the current study, this SNP was not significant [− log_10_(P-value) = 4.05]. The LD between this SNP and the most significant SNP from the current study (at 41.8 Mb on SSC7) was only 0.11. Although this is not a strong LD, it indicates that this region on SSC7 plays an important role in litter size variability.

The two genes that are located within ± 50 kb from the most significant SNP on SSC7 for both traits are *ADGRF1*—*adhesion G protein-coupled receptor F1* (SSC7:41,806,393–41,853,657 bp) and *ADGRF5*—*adhesion G protein-coupled receptor F5* (SSC7:41,640,258–41,769,286 bp). The *ADGRF1* GO term annotation relates it to transmembrane signaling receptor activity, for which *ADGRF5* is an important paralog [27]. *ADGRF1* is involved in neurodevelopment of the brain and its functions are related to the effect of docosahexaenoic acid on the brain [42, 43]. *ADGRF5* was recently reported to be involved in the prevention of paraquat-related lung injuries and to be important for the function of the respiratory system [44] and in the process of vascularization [45, 46]. Moreover, knockout mice that lack *ADGRF5* (in combination with a knockout of another gene *adhesion G protein-coupled receptor ADGRL4*) show perinatal lethality in 50% of the animals [46]. These functions can have an important effect on the blood supply to developing fetuses. Both *ADGRF1* and *ADGRF5* are expressed in human ovary, uterus, prostate, and testis tissues [27].

Another identified vQTL region for varTNB was also located on SSC7, with the most significant SNP being AX-116320493 and the candidate gene being *AP3B2*—*adaptor related protein complex 3 subunit beta 2*, which is expressed in the testis and prostate [27]. Mutations of this gene have been reported to result in neurologic disorders in humans [47].

The two other candidate genes for LnVarTNB were *GANC*—*glucosidase alpha, neutral C* for the identified vQTL on SSC1 and *AHCYL2 adenosylhomocysteinase like 2* on SSC18. In humans, *GANC* is expressed in several organs related to reproduction in females (breast, uterus, cervix, and ovary) and in males (testis, prostate, and seminal vesicle) [27]. *GANC* encodes a member of the glycosyl family, which is a key enzyme in the metabolism of glycogen and is associated with susceptibility to diabetes [48, 49]. *AHCYL2* is involved in the development of congenital heart defects in humans, and a GWAS in Chinese cattle also described it as a candidate gene associated with displacement of the abomasum [50]. It is also expressed in the ovary and uterus and in the testis and prostate [27].

The last of the most significant SNPs for varTNB was AX-116416839 located on SSC11 within the candidate gene *KATNAL1 katanin catalytic subunit A1 like 1*. This gene is associated mostly with an expression in neurons [51], but it has been shown to have a role in the regulation of the Sertoli cell microtubules, which if disturbed can cause male infertility [52]. Since the functions of the candidate genes are not directly linked with female reproduction, it would be useful to further study the existence of causative mutations underlying variability of litter size.

## Conclusions

We identified six novel genomic regions that are associated with the variability of litter size in pigs. Only one vQTL region, on SSC7, partially overlapped between the variability traits obtained with the two methods (LnVar and DHGLM) used here and, in both cases, it contained the same most significant SNP. Both our current findings and those of a previous study of the same population, provide strong evidence for a causative mutation on SCC7 for litter size variability. However, future studies based on sequence data are needed to confirm the genomic regions involved in the control of variability of litter size, since the most significant SNP on SSC7 was detected within a non-coding region. In addition, our results indicate that the LnVar and DHGLM methods that were used to estimate the variability of litter size produced phenotypically and genetically different traits, and that DHGLM yields much more accurate results. Thus, we recommend the use of DHGLM over the simpler method to study and implement selection on the variability of litter size in breeding programs.

## Supplementary Information

**Additional file 1: Figure S1.** Expected and observed − log10 P-values of SNPs associated with litter size variability defined as LnVarTNB and varTNB with inflation factor (lambda) that were estimated using the R package QQperm [74]. **Figure S2.** Manhattan plot of the genome-wide association study for total number born in the Large White pig population.

**Additional file 2: Table S1.** SNPs significantly associated with the total number born with chromosome number (SSC) and minor allele frequency (MAF).

## Data Availability

The datasets generated and/or analyzed during the current study are not publicly available because they are part of the commercial breeding program of Topigs Norsvin. They are however available upon reasonable request (contact: egbert.knol@topigsnorsvin.com).
