# Supplementary material for: Evaluation of the phenotypic and genomic background of variability based on litter size of Large White pigs
Source: Genet Sel Evol. 2022 Jan 3;54:1. doi: 10.1186/s12711-021-00692-5 (PMC8722267; doi:10.1186/s12711-021-00692-5)
Supplement: Supplementary file 1 — Additional file 1: Figure S1. Expected and observed − log10 P-values of SNPs associated with litter size variability defined as LnVarTNB and varTNB with inflation factor (lambda) that were estimated using the R package QQperm [74]. Figure S2. Manhattan plot of the genome-wide association study for total number born in the Large White pig population. [file 12711_2021_692_MOESM1_ESM.docx]

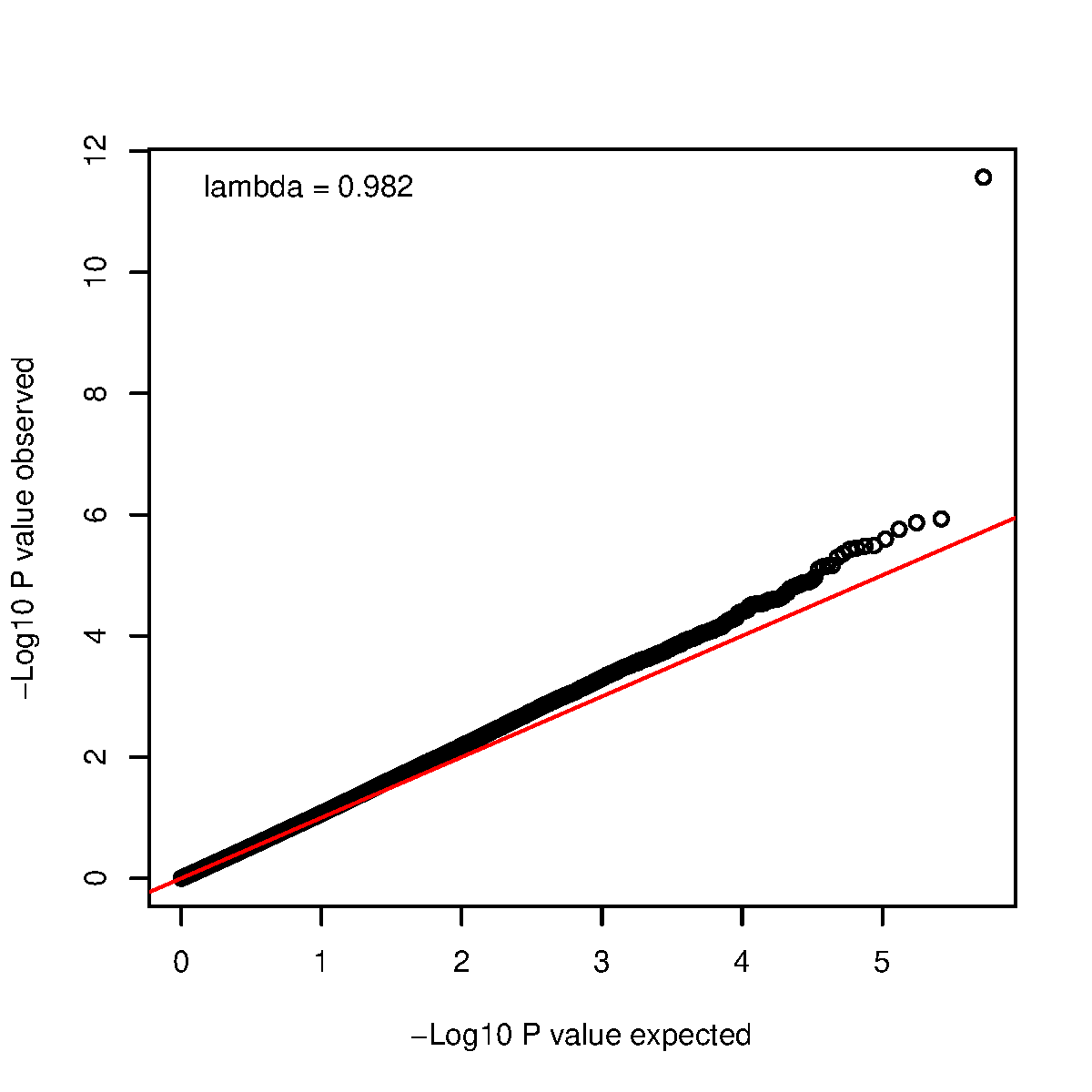

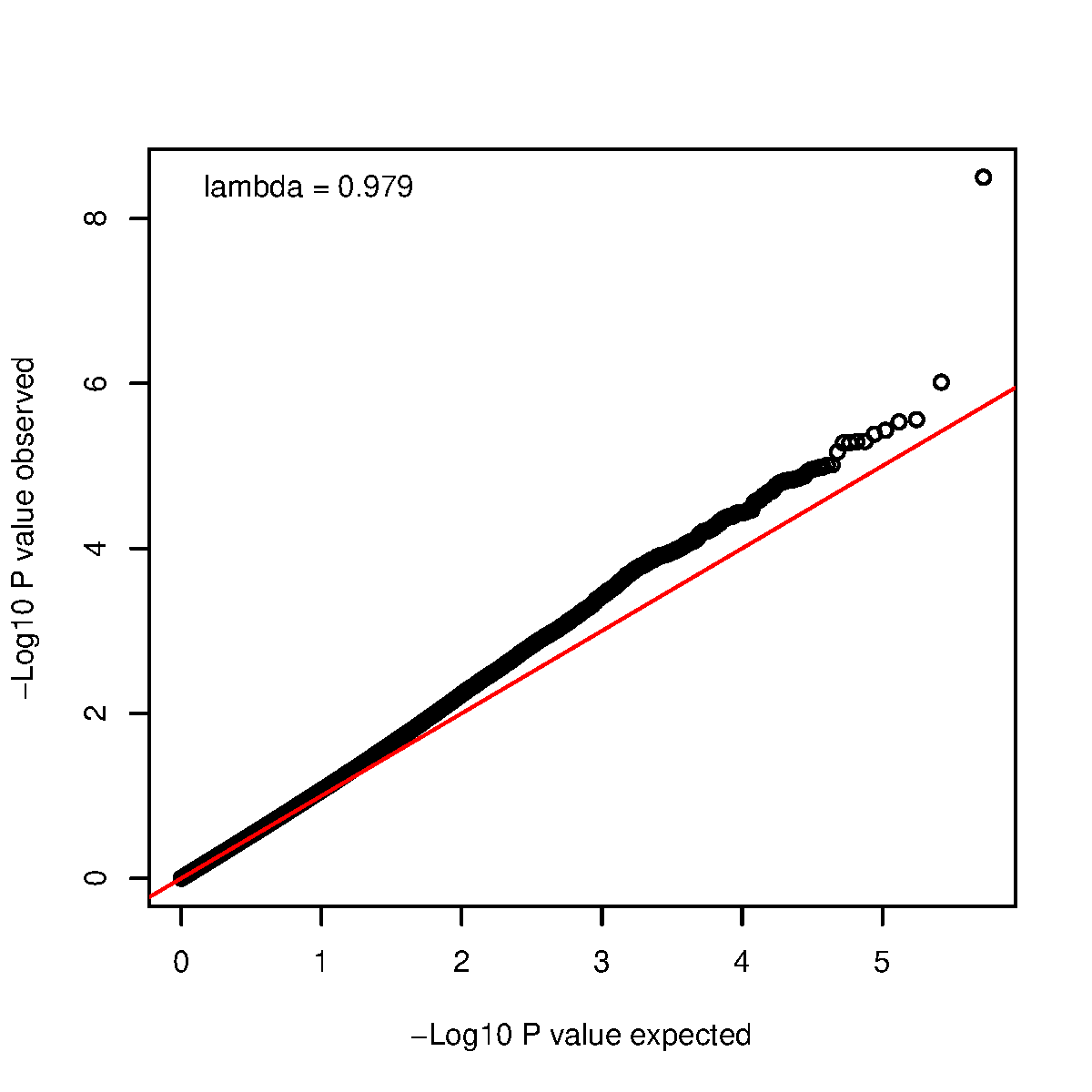


varTNB

LnVarTNB

**Figure S1.** Expected and observed –log_10_ P-values of SNPs associated with litter size variability defined as LnVarTNB and varTNB with inflation factor^a^ (lambda).

^a^Inflation factors (lambda) were estimated using the R package *QQperm* (Petrovski & Wang, 2016).


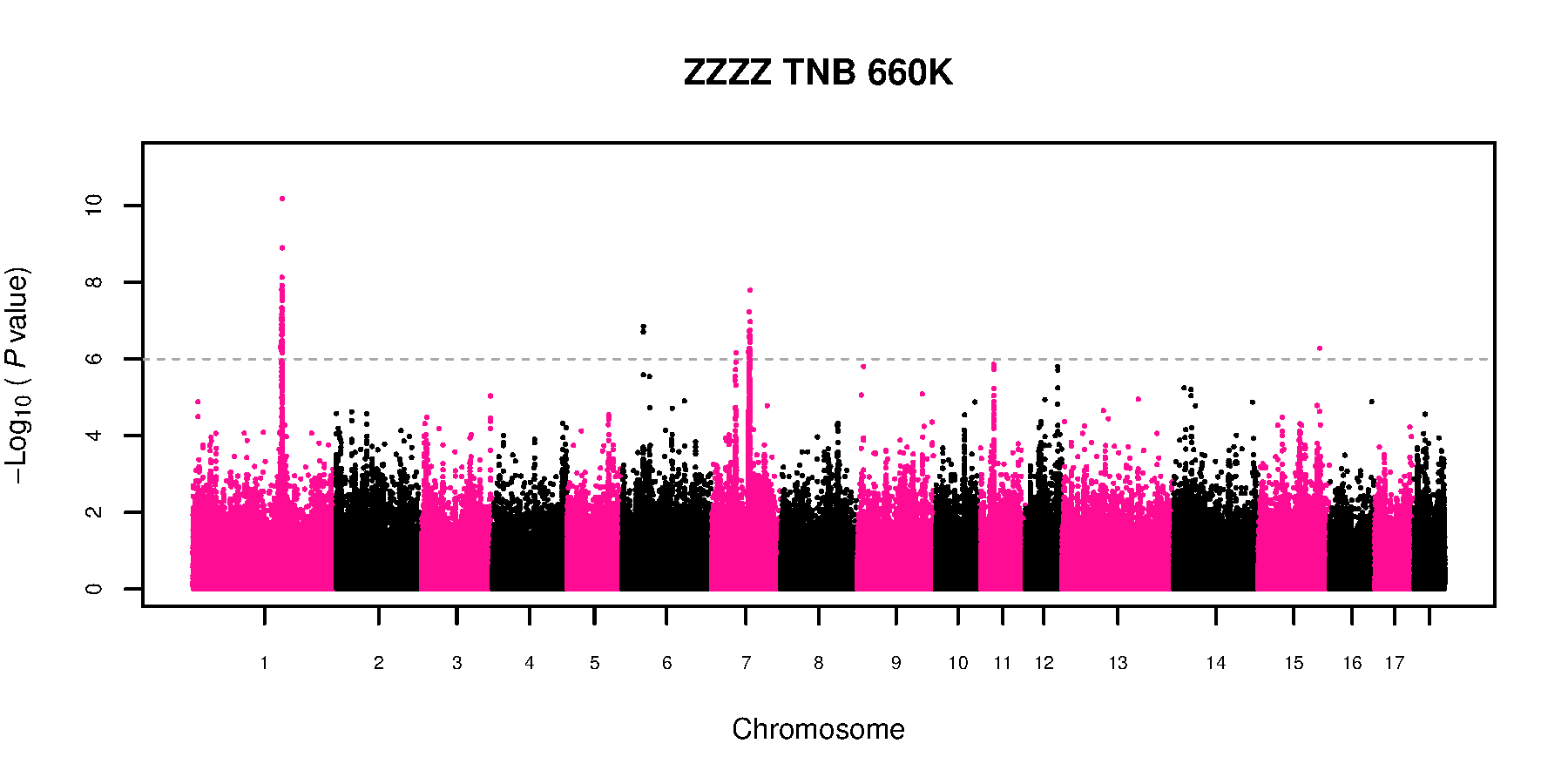


**Figure S2.** Manhattan plot of the genome-wide association study for total number born in Large White pigs population.
