# Supplementary material for: Evaluation of the phenotypic and genomic background of variability based on litter size of Large White pigs
Source: Genet Sel Evol. 2022 Jan 3;54:1. doi: 10.1186/s12711-021-00692-5 (PMC8722267; doi:10.1186/s12711-021-00692-5)
Supplement: Supplementary file 2 — Additional file 2: Table S1. SNPs significantly associated with the total number born with chromosome number (SSC) and minor allele frequency (MAF). [file 12711_2021_692_MOESM2_ESM.docx]

**Table S1.** SNPs significantly associated with the total number born with chromosome number (SSC) and minor allele frequency (MAF).

| **SSC** | **Name** | **Position (Mbp)** | **MAF** | **SNP effect** | **SE** | **Gen. var. explained** | **Phen. var. explained** | **-log_10_(p-value)** |
| --- | --- | --- | --- | --- | --- | --- | --- | --- |
| 1 | AX-116134223 | 164.74 | 0.486467 | -0.20 | 0.03 | 0.02 | 0.01 | 10.19 |
| 1 | AX-116134225 | 164.74 | 0.476091 | -0.20 | 0.03 | 0.02 | 0.01 | 8.90 |
| 1 | AX-116134044 | 164.05 | 0.492987 | -0.22 | 0.04 | 0.03 | 0.01 | 8.13 |
| 1 | AX-116134115 | 164.37 | 0.476091 | -0.21 | 0.04 | 0.03 | 0.01 | 7.91 |
| 1 | AX-116664214 | 164.38 | 0.476091 | -0.21 | 0.04 | 0.03 | 0.01 | 7.91 |
| 1 | AX-116134116 | 164.38 | 0.47646 | -0.20 | 0.04 | 0.02 | 0.01 | 7.80 |
| 1 | AX-116133958 | 163.70 | 0.489296 | -0.19 | 0.03 | 0.02 | 0.01 | 7.80 |
| 1 | AX-116134120 | 164.39 | 0.475148 | -0.20 | 0.04 | 0.02 | 0.01 | 7.78 |
| 1 | AX-116703723 | 164.03 | 0.492782 | -0.21 | 0.04 | 0.03 | 0.01 | 7.77 |
| 1 | AX-116703745 | 164.52 | 0.471703 | -0.19 | 0.03 | 0.02 | 0.01 | 7.70 |
| 1 | AX-116703754 | 164.69 | 0.475025 | -0.19 | 0.03 | 0.02 | 0.01 | 7.66 |
| 1 | AX-116134172 | 164.58 | 0.47605 | -0.20 | 0.04 | 0.02 | 0.01 | 7.63 |
| 1 | AX-116134231 | 164.76 | 0.471908 | -0.19 | 0.03 | 0.02 | 0.01 | 7.62 |
| 1 | AX-116134151 | 164.53 | 0.486795 | -0.19 | 0.03 | 0.02 | 0.01 | 7.62 |
| 1 | AX-116703753 | 164.67 | 0.475025 | -0.19 | 0.03 | 0.02 | 0.01 | 7.53 |
| 1 | AX-116133978 | 163.90 | 0.493438 | -0.20 | 0.04 | 0.02 | 0.01 | 7.34 |
| 1 | AX-116133981 | 163.89 | 0.493438 | -0.20 | 0.04 | 0.02 | 0.01 | 7.33 |
| 1 | AX-116134119 | 164.39 | 0.476829 | -0.20 | 0.04 | 0.02 | 0.01 | 7.32 |
| 1 | AX-116134007 | 163.90 | 0.493315 | -0.20 | 0.04 | 0.02 | 0.01 | 7.31 |
| 1 | AX-116134294 | 164.87 | 0.475025 | -0.20 | 0.04 | 0.02 | 0.01 | 7.31 |
| 1 | AX-116134112 | 164.36 | 0.480192 | -0.19 | 0.04 | 0.02 | 0.01 | 7.24 |
| 1 | AX-116133979 | 163.90 | 0.494915 | -0.19 | 0.04 | 0.02 | 0.01 | 7.15 |
| 1 | AX-116133987 | 163.86 | 0.493807 | -0.20 | 0.04 | 0.02 | 0.01 | 7.09 |
| 1 | AX-116134018 | 163.95 | 0.492659 | -0.20 | 0.04 | 0.02 | 0.01 | 7.06 |
| 1 | AX-116133993 | 163.84 | 0.493438 | -0.20 | 0.04 | 0.02 | 0.01 | 7.01 |
| 1 | AX-116134193 | 164.65 | 0.476665 | -0.19 | 0.04 | 0.02 | 0.01 | 7.00 |
| 1 | AX-116134047 | 164.09 | 0.493192 | -0.19 | 0.04 | 0.02 | 0.01 | 6.91 |
| 1 | AX-116133983 | 163.89 | 0.493807 | -0.20 | 0.04 | 0.02 | 0.01 | 6.91 |
| 1 | AX-116134195 | 164.66 | 0.476665 | -0.19 | 0.04 | 0.02 | 0.01 | 6.90 |
| 1 | AX-116133963 | 163.72 | 0.477813 | -0.17 | 0.03 | 0.02 | 0.01 | 6.89 |
| 1 | AX-116664216 | 164.64 | 0.478428 | -0.18 | 0.03 | 0.02 | 0.01 | 6.85 |
| 1 | AX-116134198 | 164.67 | 0.476542 | -0.19 | 0.04 | 0.02 | 0.01 | 6.84 |
| 1 | AX-116134229 | 164.76 | 0.469611 | -0.18 | 0.03 | 0.02 | 0.01 | 6.83 |
| 1 | AX-116133937 | 163.77 | 0.492823 | -0.18 | 0.04 | 0.02 | 0.01 | 6.82 |
| 1 | AX-116134183 | 164.59 | 0.451362 | -0.17 | 0.03 | 0.02 | 0.01 | 6.80 |
| 1 | AX-116134181 | 164.60 | 0.476296 | -0.18 | 0.03 | 0.02 | 0.01 | 6.78 |
| 1 | AX-116133995 | 163.83 | 0.490116 | -0.18 | 0.03 | 0.02 | 0.01 | 6.76 |
| 1 | AX-116703755 | 164.76 | 0.46916 | -0.18 | 0.03 | 0.02 | 0.01 | 6.76 |
| 1 | AX-116134185 | 164.62 | 0.475517 | -0.18 | 0.03 | 0.02 | 0.01 | 6.72 |
| 1 | AX-116134186 | 164.63 | 0.476255 | -0.18 | 0.04 | 0.02 | 0.01 | 6.65 |
| 1 | AX-116134097 | 164.30 | 0.484826 | 0.15 | 0.03 | 0.01 | 0.00 | 6.62 |
| 1 | AX-116133962 | 163.71 | 0.493274 | -0.19 | 0.04 | 0.02 | 0.01 | 6.62 |
| 1 | AX-116134175 | 164.62 | 0.476829 | -0.18 | 0.04 | 0.02 | 0.01 | 6.49 |
| 1 | AX-116134026 | 163.97 | 0.49188 | -0.18 | 0.04 | 0.02 | 0.01 | 6.48 |
| 1 | AX-116133972 | 163.80 | 0.485482 | -0.18 | 0.03 | 0.02 | 0.01 | 6.47 |
| 1 | AX-116703756 | 164.78 | 0.468791 | -0.18 | 0.04 | 0.02 | 0.01 | 6.46 |
| 1 | AX-116703713 | 163.88 | 0.498606 | 0.18 | 0.04 | 0.02 | 0.01 | 6.40 |
| 1 | AX-116134179 | 164.61 | 0.475025 | -0.18 | 0.03 | 0.02 | 0.01 | 6.40 |
| 1 | AX-116134200 | 164.67 | 0.470226 | -0.16 | 0.03 | 0.02 | 0.01 | 6.35 |
| 1 | AX-116133340 | 161.25 | 0.431471 | -0.16 | 0.03 | 0.01 | 0.00 | 6.31 |
| 1 | AX-116134114 | 164.37 | 0.495858 | -0.17 | 0.03 | 0.02 | 0.01 | 6.25 |
| 1 | AX-116133999 | 163.82 | 0.484375 | -0.18 | 0.04 | 0.02 | 0.01 | 6.22 |
| 1 | AX-116134258 | 164.78 | 0.468914 | -0.17 | 0.04 | 0.02 | 0.01 | 6.16 |
| 1 | AX-116134257 | 164.79 | 0.468914 | -0.17 | 0.04 | 0.02 | 0.01 | 6.16 |
| 6 | AX-116281134 | 40.53 | 0.0755413 | 0.24 | 0.05 | 0.01 | 0.00 | 6.85 |
| 6 | AX-116281133 | 40.53 | 0.0757874 | 0.24 | 0.05 | 0.01 | 0.00 | 6.71 |
| 7 | AX-116317698 | 38.81 | 0.170153 | 0.18 | 0.04 | 0.01 | 0.00 | 6.16 |
| 7 | AX-116322793 | 65.81 | 0.257177 | 0.21 | 0.04 | 0.02 | 0.01 | 7.79 |
| 7 | AX-116322472 | 64.39 | 0.328248 | 0.18 | 0.03 | 0.02 | 0.01 | 7.23 |
| 7 | AX-116808534 | 65.81 | 0.251599 | 0.19 | 0.04 | 0.02 | 0.01 | 6.97 |
| 7 | AX-116322691 | 65.46 | 0.260622 | 0.19 | 0.04 | 0.02 | 0.01 | 6.74 |
| 7 | AX-116630043 | 64.22 | 0.356545 | 0.17 | 0.03 | 0.02 | 0.01 | 6.73 |
| 7 | AX-116767456 | 65.45 | 0.260909 | 0.19 | 0.04 | 0.02 | 0.01 | 6.62 |
| 7 | AX-116322521 | 64.56 | 0.253855 | 0.19 | 0.04 | 0.02 | 0.01 | 6.60 |
| 7 | AX-116322799 | 65.83 | 0.287156 | 0.18 | 0.03 | 0.02 | 0.01 | 6.58 |
| 7 | AX-116322566 | 64.86 | 0.257669 | 0.19 | 0.04 | 0.02 | 0.01 | 6.55 |
| 7 | AX-116849574 | 64.86 | 0.257669 | 0.19 | 0.04 | 0.02 | 0.01 | 6.55 |
| 7 | AX-116322692 | 65.46 | 0.259391 | 0.19 | 0.04 | 0.02 | 0.01 | 6.51 |
| 7 | AX-116767457 | 65.46 | 0.259391 | 0.19 | 0.04 | 0.02 | 0.01 | 6.51 |
| 7 | AX-116322811 | 65.88 | 0.255454 | 0.19 | 0.04 | 0.02 | 0.01 | 6.47 |
| 7 | AX-116768918 | 65.71 | 0.257136 | 0.19 | 0.04 | 0.02 | 0.01 | 6.43 |
| 7 | AX-116630044 | 64.23 | 0.36122 | 0.16 | 0.03 | 0.01 | 0.00 | 6.28 |
| 7 | AX-116322568 | 64.87 | 0.256398 | 0.19 | 0.04 | 0.02 | 0.01 | 6.27 |
| 7 | AX-116322720 | 65.54 | 0.258079 | 0.19 | 0.04 | 0.02 | 0.01 | 6.25 |
| 7 | AX-116768917 | 65.24 | 0.257915 | 0.19 | 0.04 | 0.02 | 0.01 | 6.19 |
| 7 | AX-116322027 | 62.22 | 0.183071 | 0.17 | 0.04 | 0.01 | 0.00 | 6.18 |
| 7 | AX-116322770 | 65.71 | 0.255865 | 0.19 | 0.04 | 0.02 | 0.01 | 6.16 |
| 7 | AX-116322565 | 64.86 | 0.255536 | 0.19 | 0.04 | 0.02 | 0.01 | 6.16 |
| 7 | AX-116849577 | 65.24 | 0.25771 | 0.19 | 0.04 | 0.02 | 0.01 | 6.11 |
| 7 | MARC0067183 | 65.49 | 0.257013 | 0.19 | 0.04 | 0.02 | 0.01 | 6.10 |
| 7 | AX-116322413 | 64.09 | 0.326115 | 0.17 | 0.03 | 0.01 | 0.01 | 6.08 |
| 7 | AX-116849576 | 65.22 | 0.257628 | 0.18 | 0.04 | 0.02 | 0.01 | 6.08 |
| 7 | AX-116760738 | 64.57 | 0.257669 | 0.18 | 0.04 | 0.01 | 0.01 | 6.02 |
| 15 | AX-116555220 | 125.20 | 0.189592 | 0.19 | 0.04 | 0.01 | 0.00 | 6.28 |
